# The diaphragm-intercostal index for predicting weaning failure in mechanically ventilated patients: a prospective cohort study

**DOI:** 10.3389/fmed.2026.1823331

**Published:** 2026-06-12

**Authors:** Lei Zhang, Zhijia Zhao, Yaling Wu, Ning Zhu, Shiyi Liu, Jingjing Zhou, Guozhong Chen, Wanjun Yu

**Affiliations:** 1 Department of Respiratory and Critical Care Medicine, The Affiliated People’s Hospital of Ningbo University, Ningbo, Zhejiang, China; 2 Medical Record Statistics Room, The Affiliated People’s Hospital of Ningbo University, Ningbo, Zhejiang, China; 3 Child Rehabilitation Division, Ningbo Rehabilitation Hospital, Ningbo, Zhejiang, China; 4 Department of Respiratory and Critical Care Medicine, The First Affiliated Hospital of Ningbo University, Ningbo, Zhejiang, China; 5 Department of Critical Care Medicine, The Affiliated People’s Hospital of Ningbo University, Ningbo, Zhejiang, China; 6 Ningbo Municipal Hospital of Traditional Chinese Medicine (TCM), Affiliated Hospital of Zhejiang Chinese Medical University, Ningbo, Zhejiang, China

**Keywords:** critical care, diaphragm-intercostal index, mechanical ventilation, ultrasonography, weaning failure

## Abstract

**Background:**

Accurately predicting the risk of weaning failure is crucial for optimizing clinical decision-making and improving patient prognosis. Despite widespread use, the predictive value of diaphragm ultrasound remains controversial, and the compensatory role of auxiliary respiratory muscles has been overlooked. This study aims to evaluate whether the diaphragm-intercostal index (DII), a novel ultrasound index for joint assessment of diaphragm and intercostal muscle function, can predict weaning failure more accurately than traditional parameters.

**Methods:**

This prospective single-center cohort study enrolled invasive mechanical ventilation patients (≥48 h) ready for weaning. Ultrasound was performed 30 min into the spontaneous breathing trial (SBT) (pre-extubation, for primary predictive analysis) and 24 h post-extubation (for exploratory descriptive analysis only). Measurements included diaphragmatic excursion (DE), diaphragm thickening fraction (TFdi), and intercostal muscle thickening fraction (TFic). DII was calculated as TFic/TFdi. The primary outcome was weaning failure, defined as SBT failure or need for therapeutic non-invasive ventilation (NIV), reintubation, or death within 48 h post-extubation. Sensitivity analysis was performed after excluding SBT failure patients to assess DII’s performance in predicting true post-extubation failure. Lasso-logistic regression and ROC curves assessed predictive performance.

**Results:**

Of 107 patients, 81 weaned successfully and 26 failed. Among failures, 5 (19.23%) had SBT failure, 8 (30.77%) required reintubation, 11 (42.31%) required therapeutic NIV, and 2 (7.69%) died. DII’s AUC was 0.952 (95% CI: 0.907–0.998), significantly outperforming RSBI (AUC = 0.789) and TFdi (AUC = 0.830). The optimal DII cutoff was >0.485 (sensitivity 80.8%, specificity 97.5%). In the sensitivity analysis excluding SBT failures, DII’s AUC for predicting true post-extubation failure remained high at 0.942 (95% CI: 0.887–0.996), and DeLong test showed no significant difference from the primary analysis (*p* = 0.779).

**Conclusion:**

DII can effectively quantify the compensation of the auxiliary respiratory muscles relative to the diaphragm, showing favorable predictive performance for predicting weaning failure, strikingly superior to traditional parameters. However, validation in large-scale, multi-center studies is needed, particularly after standardizing SBT protocols with positive end-expiratory pressure (PEEP) as per guidelines, and in populations including obese and heart failure patients.

## Introduction

Mechanical ventilation (MV) is a crucial life support technique applied in the intensive care unit (ICU). However, safe and timely extubation from MV remains a significant clinical challenge in critical care medicine (1). Although patients can pass the spontaneous breathing test (SBT), up to 10–15% of patients still experience a failure of extubation from MV. This significantly augments the risk of ventilator-associated complications and mortality (2–5), highlighting the urgent demand for accurate and objective bedside prediction tools.

Dysfunction of the diaphragm, an important cause of failure of extubation from MV, exists in 30–60% mechanically ventilated patients (6, 7). Bedside diaphragmatic ultrasonography has been adopted in clinical practice to forecast extubation outcomes (8–10), but its predictive efficacy remains controversial (11), indicating that a single assessment of the diaphragm function may have limitations. In the context of diaphragm dysfunction or respiratory load increase, the body needs to mobilize auxiliary respiratory muscles (like intercostal muscles) for compensation (12–14). Patients with diaphragmatic weakness are often characterized by an elevation in the parasternal intercostal muscle-thickening fraction (TFic) (12, 14, 15), providing a new insight into evaluating the compensatory mechanism of respiratory muscles. Joint assessment of the diaphragm and auxiliary respiratory muscles has gradually gained attention. The TFic/TFdi ratio has shown a prediction potential for extubation failure, and its elevation hints an imbalanced state of overcompensated auxiliary respiratory muscles and relative insufficient diaphragm function (12, 14). However, this indicator has not been fully validated in prospective studies.

Based on this, this study proposes the diaphragm-intercostal index (DII), defined as TFic/TFdi, as a novel defined comprehensive indicator, which lies in its dynamic quantification of the balance between auxiliary respiratory muscle compensation and main inspiratory muscle function. We assume that DII can predict the extubation outcomes more accurately than traditional indicators (such as P0.1, RSBI) or a single parameter, providing an objective clinical assessment tool reflecting the compensatory potential of the entire respiratory muscle system.

## Methods

### Study participants and design

A prospective cohort study was conducted in the ICU of a university-affiliated hospital between January 2020 and December 2023. The research programme was ratified by the Ethics Committee is The Affiliated People’s Hospital of Ningbo University (approval No. 2019-011) and prospectively registered in the National Medical Research Registration & Filing Information System (registration no. MR-33-20-000113). All patients or their legal representatives signed a written informed consent form.

### Study participants

Inclusion criteria: (1) Age ≥ 18 years old; (2) patients who received invasive MV for at least 48 h; (3) meets the criteria for a SBT. Rationale for inclusion of COPD patients: In patients with chronic obstructive pulmonary disease (COPD), diaphragm function is often affected, and they may rely more on accessory respiratory muscles (e.g., intercostal muscles) for compensation. Based on this characteristic, eligible COPD patients were enrolled in this study to explore the predictive performance of DII in this population. The features of this population may help to examine whether DII can reflect compensatory changes in accessory respiratory muscle activity.

Exclusion criteria: (1) the presence of known diaphragmatic lesions or neuromuscular diseases (Guillain-Barré syndrome, myasthenia gravis, etc.); (2) severe underlying cardiac diseases (such as severe heart failure, unstable angina, etc.) — these were excluded to minimize confounding, as heart failure independently influences weaning outcomes; (3) recent (within 3 months) history of chest and abdominal surgery or severe chest trauma that affects ultrasound evaluation; (4) pathological obesity (BMI ≥ 35 kg/m^2^) — due to inadequate ultrasound image quality for reliable diaphragm and intercostal measurements; (5) massive pleural effusion; (6) history of pleural fixation; (7) tracheotomy status; (8) the utilization of neuromuscular blocking drugs within 48 h before extubation; (9) patients who received extracorporeal membrane oxygenation (ECMO) treatment; (10) pregnant and lactating women, as well as patients with end-stage tumors.

**Figure 1:**
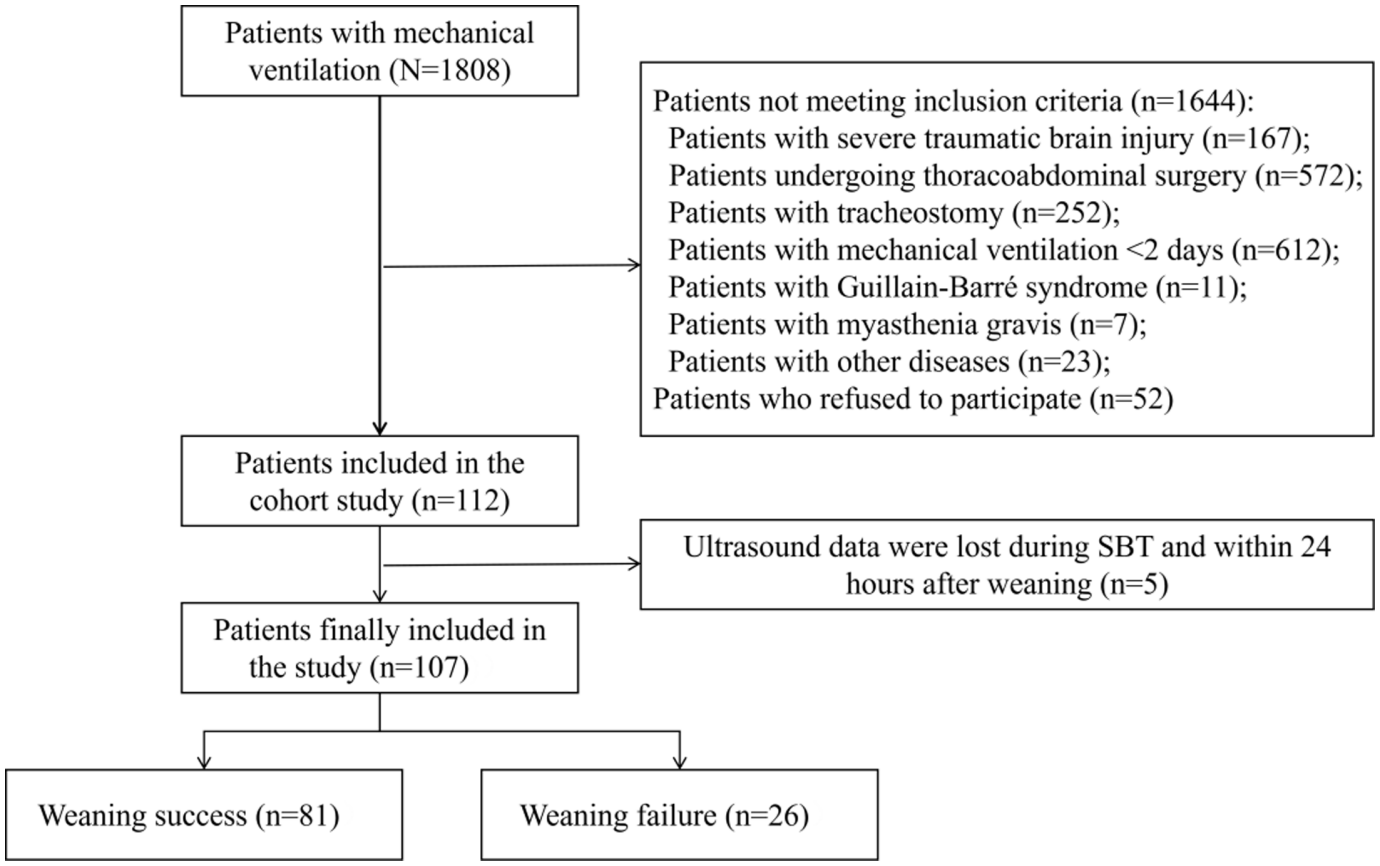
Study schematic diagram. SBT, spontaneous breathing test.

From 1,808 patients undergoing extubation after invasive MV, 107 patients conformed to all inclusion criteria and ultimately completed the entire research process (Figure 1).

### Research procedure

#### SBT and extubation outcomes

Referring to international consensus (16, 17), the criteria for extubation preparation included: (1) improvement in the primary diseases for MV; (2) fraction of inspired oxygen (FiO2) ≤ 0.50; (3) positive end-expiratory pressure (PEEP) ≤ 5–8 cm H2O; (4) RR ≤ 30 breaths/min, tidal volume (VT) ≥ 5 mL/kg; (5) ratio of arterial partial pressure of oxygen (PaO2) to FiO2 ≥ 150; (6) pH > 7.25; (7) stable hemodynamics, no need for or low dose of vasoactive drugs (norepinephrine or alternatives ≤ 0.1 μg/kg/min); (8) conscious and no use of anesthetic sedatives; (9) less airway sputum—objectively defined as endotracheal suctioning frequency <2 times per hour or total suctioned volume <20 mL over the preceding 4 h; (10) protective cough reflex.

Patients meeting extubation preparation criteria underwent a SBT using pressure support ventilation (PSV) mode. The PSV parameters were set as follows (18): pressure support 8 cmH₂O, PEEP 0 cmH₂O, inspiratory/expiratory trigger sensitivity 2 L/min, inspiratory rise time 0.2 s, FiO₂ adjusted to maintain SpO₂ ≥ 95%. The decision to use 0 cmH₂O PEEP, though deviating from current guidelines (which typically recommend 5–8 cmH₂O), was intentional: it maximally unloads the inspiratory muscles, thereby unmasking subclinical diaphragm dysfunction and compensatory recruitment of accessory muscles—the core pathophysiological concept underlying the DII. This approach enhances the sensitivity of the DII to detect respiratory muscle imbalance, but we acknowledge its impact on diaphragmatic loading and discuss it in the Limitations section. The SBT lasted 30 min (max 120 min), with FiO₂ ≤ 0.5.

#### Definition of extubation outcome

Extubation success: after successful SBT, the attending physician (blinded to ultrasound measurements) removed the endotracheal tube, and the patient maintained spontaneous breathing for ≥48 h without ventilator support.

Extubation failure: (a) SBT failure; or (b) successful SBT but requiring NIV, reintubation, or death within 48 h after extubation.

For the primary analysis, all patients meeting either (a) or (b) were classified as weaning failure. We performed a sensitivity analysis excluding the 5 patients who failed SBT (i.e., analyzing only patients with successful SBT) to determine whether DII remained predictive of true post-extubation failure.

Based on the extubation outcomes, patients were classified into extubation success and extubation failure groups.

#### Ultrasound image acquisition and measurement

Functional parameters of the diaphragm and intercostal muscles were recorded by bedside ultrasound 30 min after SBT (pre-extubation, for primary predictive analysis) and 24 h after extubation (for exploratory descriptive analysis only). All ultrasound images were collected using Sonosite Series S-ICUTM ultrasound instrument (USA) by a physician with at least a 5-year experience of critical care ultrasound (blinded to clinical data and patient grouping). The patient was kept in a semi-reclining position (30–45°), and the right diaphragm was mainly assessed.

**Figure 2:**
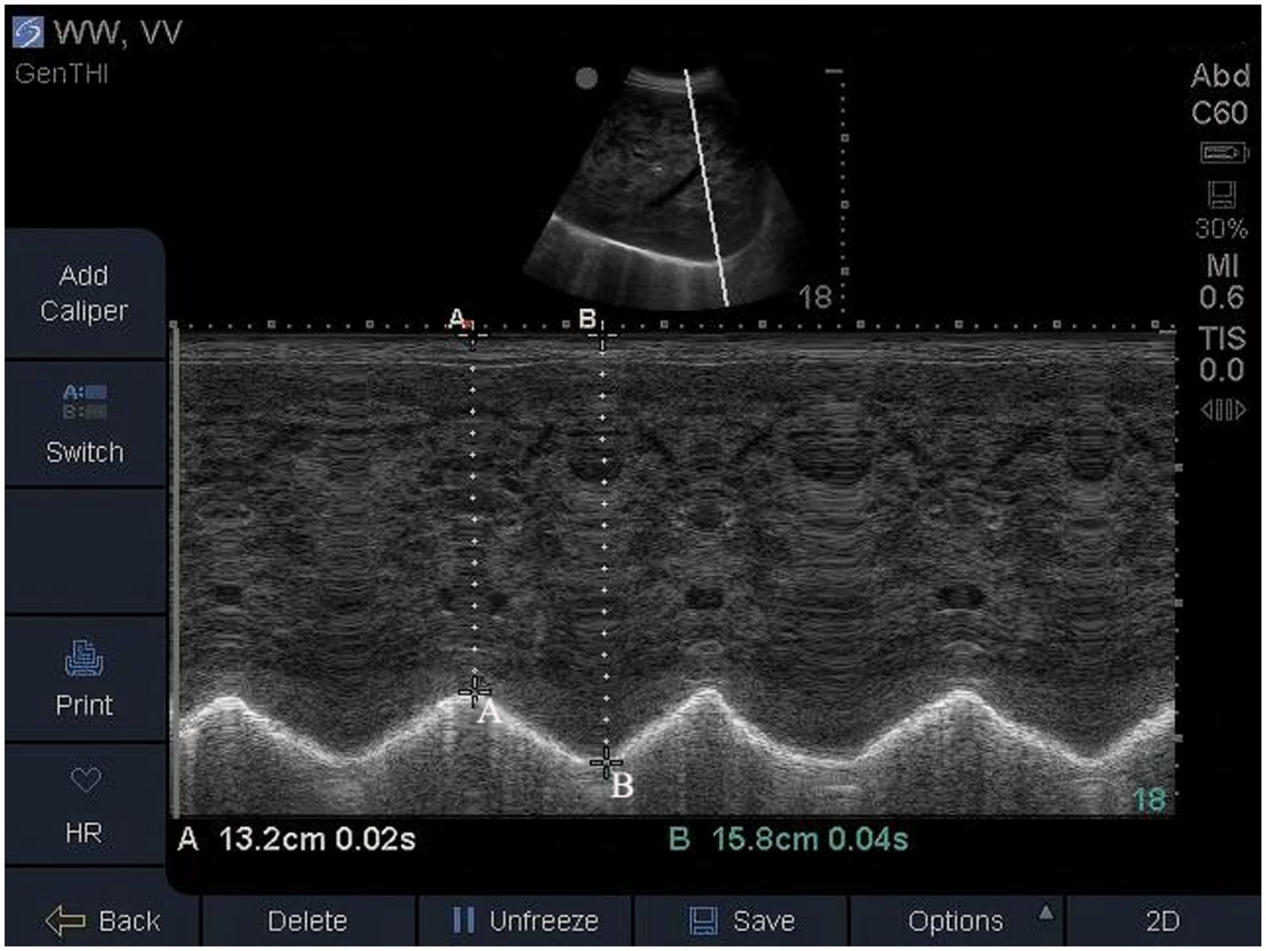
Measurement of DE, respiratory diaphragmatic movement curve in M-mode, DE = amplitude of end-inspiratory diaphragm movement (BB)-amplitude of end-expiratory diaphragm movement (AA).

DE: a convex-array probe (3.5 MHz) was put in the subcostal region on the right midline of the clavicle to display the liver-diaphragm interface and then switched to M-mode. With the angle between the sampling line and the diaphragm less than 30°, the amplitude of diaphragm movement was measured under eupnea (Figure 2).

**Figure 3:**
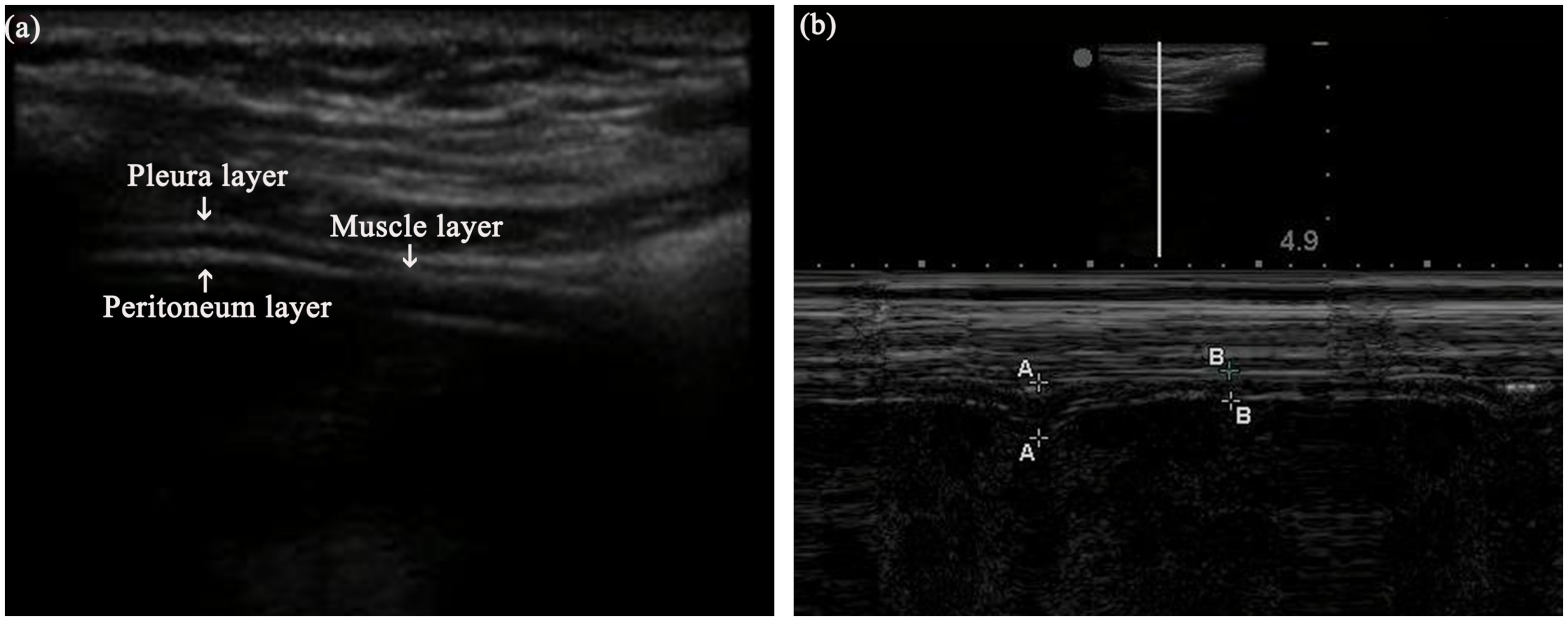
Measurement of diaphragm thickness, **(a)**: B ultrasound manifestation of diaphragm: “three-line” structure indicated by the arrows; **(b)** the respiratory diaphragm thickness change curves in the M-mode, AA: end-inspiratory diaphragm thickness (Tdi^ins^), BB: end-expiratory diaphragm thickness (Tdi^exp^).

Diaphragm thickness and TFdi: a high-frequency linear-array probe (10 MHz) was placed between the 8th and 10th intercostal space along the midaxillary line, showing a “three-line” structure of the diaphragm (Figure 3a). The end-inspiratory diaphragm thickness (Tdi^ins^) and end-expiratory diaphragm thickness (Tdi^exp^) were measured under M-mode ultrasound (Figure 3b). TFdi calculation formula: (Tdi^ins^ – Tdi^exp^)/Tdi^exp^ × 100%.

All measurements were conducted for 3 consecutive respiratory cycles, from which the values obtained were averaged.

**Figure 4:**
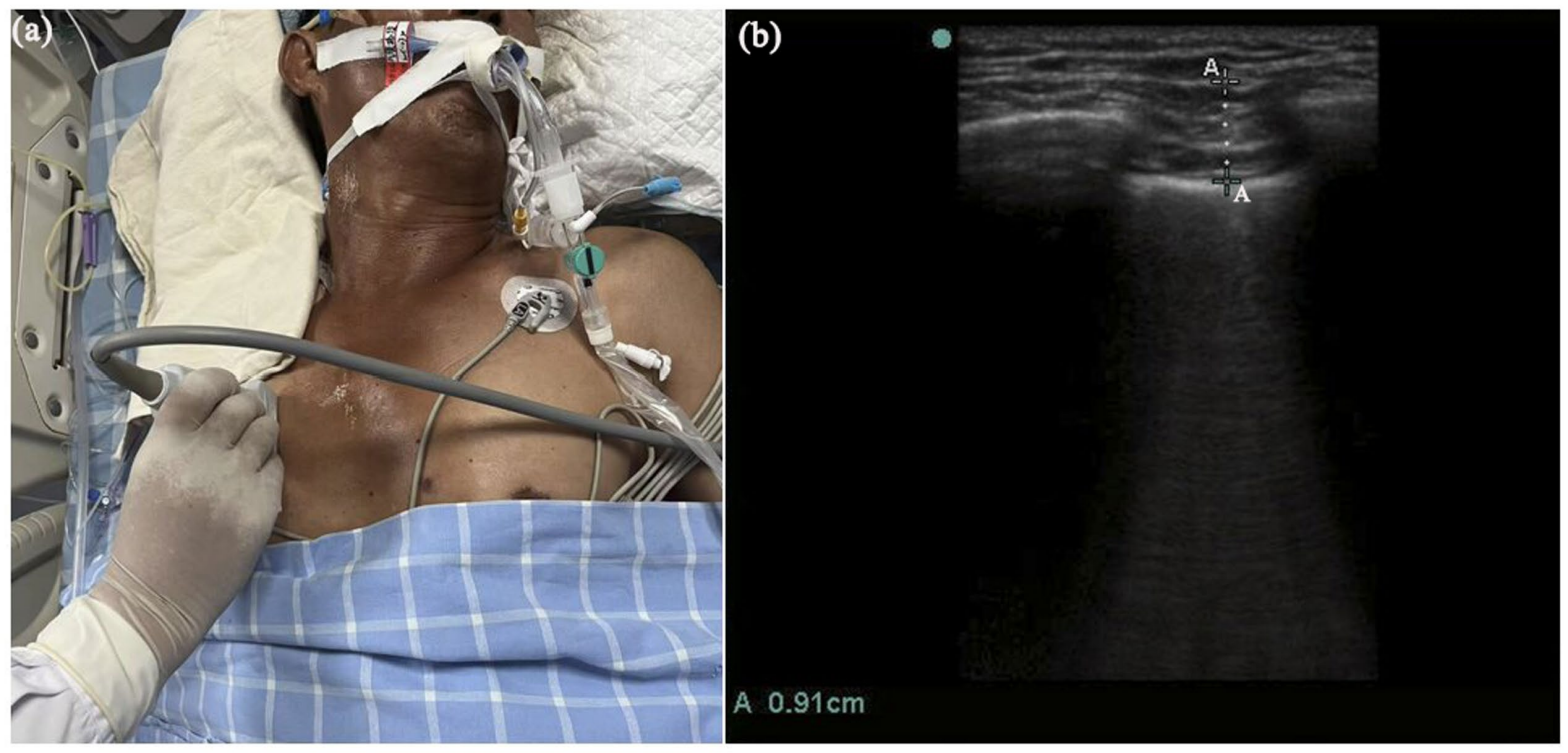
Schematic diagram of intercostal muscle ultrasound and measurement, **(a)** bedside intercostal muscle ultrasound for the patient; **(b)** Intercostal muscle ultrasound image (AA).

Ultrasound of intercostal muscles: with the patients in the supine position, a linear-array probe (10–15 MHz) was vertically placed on the second intercostal space near the sternum, showing the intercostal muscles between adjacent ribs (Figure 4). The end-inspiratory intercostal muscle thickness (Tic^ins^) and end-expiratory intercostal muscle thickness (Tic^exp^) were measured under M-mode ultrasound. TFdi calculation formula: (Tic^ins^−Tic^exp^)/Tic^exp^ × 100%. The average values were obtained from repeated measurements for 3 consecutive respiratory cycles.

DII calculation: the core indicator defined here as TFic/TFdi. This ratio aims to quantify the compensatory activity of the auxiliary respiratory muscles (intercostal muscles) relative to the main inspiratory muscles (diaphragm).

To evaluate the consistency of the measurers themselves, images of 20 patients were randomly selected by the same physician, and repeated measurements were made after a 24-h interval, and lastly, the intra-class correlation coefficient (ICC) was computed.

#### Outcome measures

Demographics, disease characteristics, MV parameters, disease severity score (APACHE II, SOFA), vital signs, blood gas analysis results, rapid shallow breathing index (RSBI), airway occlusion pressure (P0.1), and 28-day mortality were recorded. Additionally, ultrasound indicators (DE, TFdi, TFic, DII) of patients at 30 min of SBT and 24 h post extubation were compared between the two groups.

#### Sample size estimation

Based on previous research data (19, 20), we assumed that the predictive performance of DII (AUROC) was improved by about 0.12 as compared to traditional indicators (TFdi, DE). With *α* = 0.05 (two-sided) and *β* = 0.2 (test power of 80%), sample size estimation was performed using PASS2021 software. Accordingly, the minimal requisite sample size was 86 cases. Considering a dropout rate of approximately 10%, it was planned to enroll at least 93 patients. Lastly, 107 qualified cases completed the whole process with a sufficient sample size.

#### Statistical analysis

Statistical analysis was implemented using SPSS (27.0) and R (v.4.3.0) software. Measurement data that conformed to normal distribution were expressed as mean ± standard deviation (x̄ ± s), with a two independent samples *t*-test applied for inter-group comparisons; skewed measurement data, represented by median (interquartile range) [M (P_25_, P_75_)], were compared by the Mann–Whitney U test. Enumeration data were expressed in frequency (percentage) and compared through the χ^2^ test or Fisher’s exact test. Pearson or Spearman correlation analysis was applied to assess the correlation between ultrasound indicators and parameters of respiratory mechanics. The larger the absolute value of the correlation coefficient r, the higher the correlation. A positive R value indicated a positive correlation, while a negative value suggested a negative correlation. Lasso-logistic regression analysis was utilized to screen modeling factors, effectively avoiding overfitting. The optimal penalty parameter (*λ*) was screened by 10-fold cross-validation, and variables with λ = 0 were removed from the model. Based on the selected predictive factors, a nomogram model for predicting the risk of extubation failure was generated. In the nomogram, the regression coefficients corresponding to risk factors in the model were proportionally converted into 0–100 points. The total score of a patient was obtained by summing the scores of all factors. The predictive performance of various indicators (P0.1, RSBI, DE, TFdi, TFic, DII) for extubation failure was analyzed with the application of receiver operating characteristic (ROC) curves, yielding the area under the curve (AUC). The optimal cutoff value was determined according to the Youden index, and the cutoff value, sensitivity, and specificity were also obtained. The differences among AUC values were compared using the DeLong test. All tests were two-sided, with *p* < 0.05 indicating statistically significant differences.

All post-extubation ultrasound data (24 h) were used solely for exploratory purposes to compare the trajectory of respiratory muscle function between groups. Clinical prediction was based exclusively on pre-extubation (30-min SBT) measurements.

## Results

### Comparisons of baseline clinical data

The enrolled mechanically ventilated patients (*n* = 107) included 81 extubation success cases (75.70%, 44 males and 37 females), with an average age of (73.16 ± 12.71) years, and 26 extubation failure cases (24.30%, 17 males, 9 females), with an average age of (75.30 ± 11.32) years. Of the 26 weaning failures, 5 (19.23%) had SBT failure, 8 (30.77%) required reintubation within 48 h, 11 (42.31%) required non-invasive ventilation, and 2 (7.69%) died. No statistically significant changes were detected in age, gender, BMI, primary diseases for MV, etiological causes for MV, APACHE-II score, and 28-day mortality between the aforementioned two groups (*p* > 0.05). The extubation failure group exhibited remarkably extended duration of MV, length of ICU day, and total length of day (*p* < 0.001) and an increased SOFA score, as compared to the extubation success group (*p* < 0.05), as shown in Table 1.

**Table 1:** Comparisons of baseline clinical data between the weaning success and failure groups.

Variable	Weaning success (*n* = 81)	Weaning failure (*n* = 26)	*t*/*χ^2^*/*Z*	*p*
Age/years	72 (61–79)	66 (57–75)	0.341	0.079
Gender/*n*(%)			2.421	0.120
Male	44 (54.32)	17 (65.38)		
Female	37 (45.70)	9 (34.62)		
BMI (kg·m^−2^)	22.16 ± 2.31	24.11 ± 1.89	−1.322	0.112
Primary diseases for MV/*n*(%)			0.046	0.996
Severe pneumonia	27 (33.33)	7 (26.92)		
Acute heart failure	18 (22.22)	5 (19.23)		
AECOPD	15 (18.52)	4 (15.38)		
Septic shock	8 (9.90)	4 (15.38)		
Poisoning	6 (7.40)	2 (7.69)		
Cardiac and respiratory arrest	4 (4.94)	2 (7.69)		
Others	3 (3.70)	2 (7.69)		
Etiological causes for MV/*n*(%)			0.192	0.882
Pulmonary cause	42 (51.85)	11 (42.31)		
Extra-pulmonary cause	39 (48.15)	15 (57.69)		
Duration of MV at the beginning of SBT/d	6.2 (4.5–9.5)	9.2 (6.7–11.3)	−5.691	<0.001
Length of ICU day/d	8 (5–14.5)	16 (9–21)	−6.730	<0.001
Total length of day/d	17 (14–21)	25 (16–34)	−4.539	<0.001
28-day mortality/*n*(%)	11 (13.58)	7 (26.92)		0.611
APACHE-II score/points	19.5 (15–23)	23.0 (15.5–32)	−1.585	0.113
SOFA score/points	7.0 (3.0–9.8)	9.0 (7–14)	−2.384	0.037

BMI, body mass index; MV: mechanical ventilation; SBT, spontaneous breathing test; APACHE-II score, acute physiology and chronic health evaluation II score; SOFA score, sequential organ failure assessment score.

### Clinical and ultrasound data during extubation period

**Figure 5:**
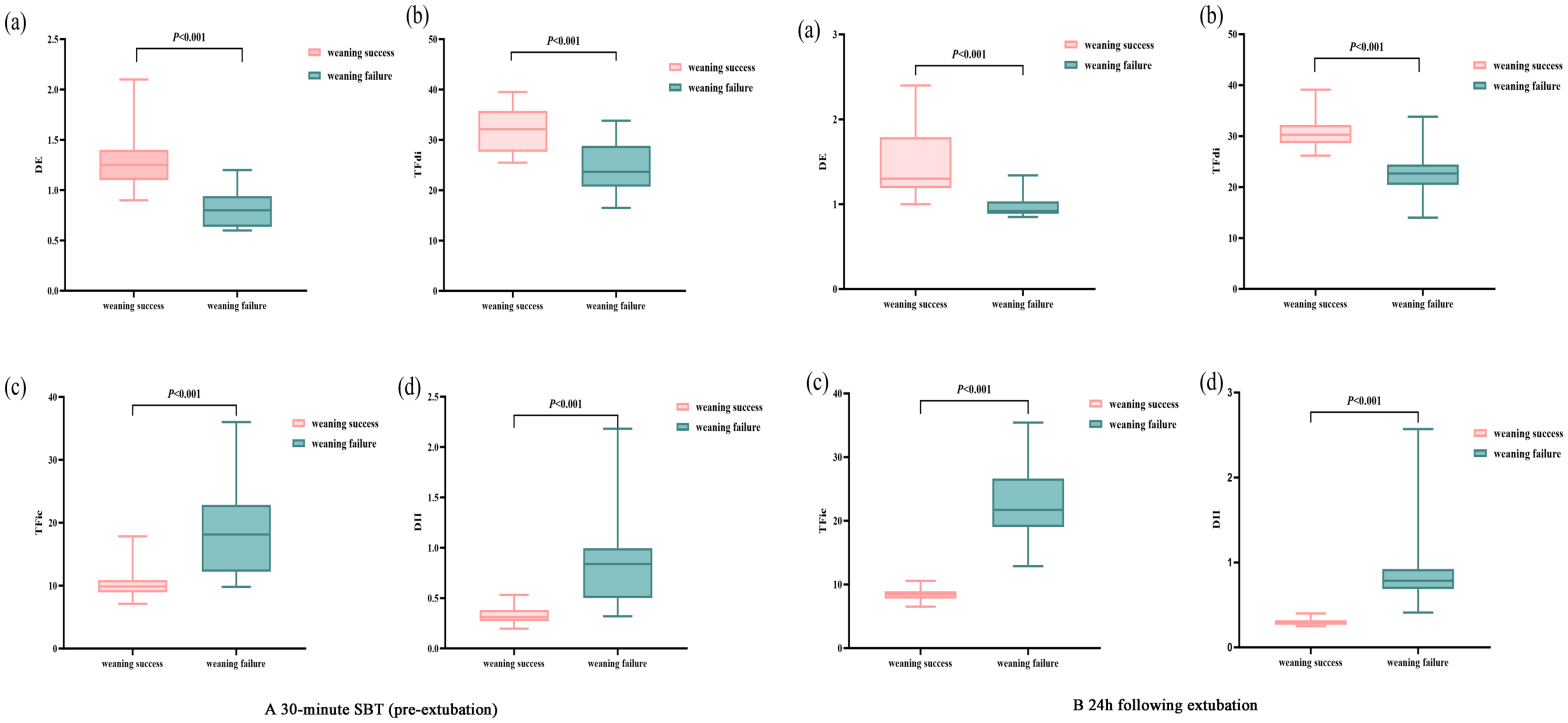
Comparisons of respiratory muscle ultrasound indicators between the weaning success and failure groups at 30 min of SBT (pre-extubation) and 24 h following extubation. **A(a)**, a comparative analysis of DE at 30 min of SBT; **A(b)**, a comparative analysis of TFdi at 30 min of SBT; **A(c)**, a comparative analysis of TFic at 30 min of SBT; **A(d)**, a comparative analysis of DII at 30 min of SBT; **B(a)**, a comparative analysis of DE 24 h following extubation; **B(b)**, a comparative analysis of TFdi 24 h following extubation; **B(c)**, a comparative analysis of TFic 24 h following extubation; **B(d)**, a comparative analysis of DII 24 h following extubation; DE, diaphragmatic excursion; TFdi, diaphragmatic thickening fraction; TFic, intercostal muscle-thickening fraction; DII, diaphragm-intercostal index.

No statistically remarkable difference was detected regarding clinical characteristic indicators such as vital signs, blood gas analysis, cardiac function indicators, biochemical indicators, and inflammatory indicators between the extubation success and failure groups during extubation after SBT (*p* > 0.05). The extubation failure group showed significantly higher pH value, PS, and PEEP (*p* < 0.05) as well as sharply higher P0.1 and RSBI values (*p* < 0.001) than the extubation success group, as presented in Table 2. At 30 min of SBT, the DE and TFdi were dramatically lower (*p* < 0.001) while TFic and DII were sharply higher (*p* < 0.001) in the extubation failure population than in the extubation success population (Figure 5); at 24 h after extubation, remarkably lower DE and TFdi (*p* < 0.001) but considerably higher TFic and DII ratios (*p* < 0.001) were observed in the extubation failure population than in the extubation success population (Figure 5). The post-extubation (24 h) comparisons presented in Table 2 and Figure 5 are exploratory and not intended for clinical prediction.

**Table 2:** Comparison of clinical and ultrasound parameters between the two groups during the peri-weaning period.

Variable	Weaning success (*n* = 81)	Weaning failure (*n* = 26)	*t*/*Z*	*P*
HR (beats/min, mean ± SD)	79.02 ± 12.40	80.43 ± 13.19	0.342	0.733
RR (breaths/min, mean ± SD)	16.90 ± 2.90	17.50 ± 3.91	−0.316	0.057
MAP (mmHg, mean ± SD)	89.12 ± 11.34	96.05 ± 12.18	−2.004	0.072
SpO_2_ (%, mean ± SD)	98.67 ± 0.65	98.44 ± 0.71	1.666	0.098
Body temperature/°C	36.9 ± 0.70	37.01 ± 0.61	1.025	0.312
LVEF (%, mean ± SD)	60.20 ± 3.44	56.28 ± 5.71	−3.008	0.601
BNP/(ng/L)	130 (36.7–397.5)	237 (133.5–726.8)	−1.613	0.107
Serum albumin/(g/L)	38.03 ± 5.43	36.21 ± 4.47	3.291	0.812
Pre-albumin/(mg/L)	289.3 ± 47.9	275.9 ± 50.3	1.249	0.214
Serum (Cr/umol/L)	75 (57–102.4)	54 (36–185)	−0.476	0.634
WBC/(×10^9^/L)	9.52 ± 1.71	10.07 ± 2.56	−0.537	0.498
Serum (CRP/(mg/L))	55.32 ± 12.45	57.91 ± 11.71	−0.811	0.472
PCT/(ng/L)	1.02 ± 0.42	0.98 ± 0.27	−0.121	0.778
Blood gas analysis				
pH (mean ± SD)	7.41 ± 0.05	7.42 ± 0.02	0.884	0.021
Partial pressure of O_2_/(mmHg, mean ± SD)	97.4 ± 17.23	92.9 ± 14.56	1.233	0.297
OI (mmHg, mean ± SD)	297.90 ± 23.16	312.50 ± 19.58	0.591	0.423
Partial pressure of CO_2_ (mmHg, mean ± SD)	37.49 ± 6.33	38.43 ± 7.51	0.535	0.595
Base excess/(mmol/L)	1.95 ± 1.12	3.10 ± 1.88	−1.724	0.093
Lactic acid (mmol/L, mean ± SD)	1.33 ± 0.21	1.34 ± 0.22	0.230	0.817
MV parameters				
Tidal volume/mL	440 ± 80.23	398 ± 77.12	−1.788	0.719
PS/cmH_2_O	12 (11, 13)	14 (12.75, 15)	−5.076	<0.001
P0.1	1.8 (1.6, 2.2)	4.2 (2.95, 5.625)	−6.649	<0.001
PEEP/cmH_2_O	3 (2, 4)	4 (3.75, 4.25)	−3.527	<0.001
RSBI/breaths/((min.L), mean ± SD)	81.68 ± 18.36	105.27 ± 16.07	−7.654	<0.001
At 30 min of the 1st SBT (pre-extubation)				
DE/cm	1.25 (1.1, 1.4)	0.80 (0.64, 0.94)	−6.629	<0.001
TFdi/%	31.65 (27.63, 35.24)	25.29 (20.73, 30.93)	−4.369	<0.001
TFic/%	9.88 (8.95, 10.88)	19.19 (13.43, 22.84)	−6.462	<0.001
DII	0.32 (0.27, 0.38)	0.82 (0.53, 1.00)	−6.690	<0.001
24 h after the 1st extubation				
DE/cm	1.30 (1.19, 1.79)	0.92 (0.89, 1.03)	−6.536	<0.001
TFdi/%	30.27 (28.64, 32.21)	22.66 (20.44, 24.43)	−6.803	<0.001
TFic/%	8.41 (7.80, 8.94)	21.73 (19.03, 26.65)	−7.649	<0.001
DII	0.28 (0.27, 0.32)	0.79 (0.69, 0.92)	−7.670	<0.001

OI, oxygenation index; HR, heart rate; RR, respiratory rate; MAP, mean arterial pressure; RSBI: rapid shallow breathing index; P0.1, airway occlusion pressure at 100 msec; PEEP, positive end-expiratory pressure; PS, pressure support; PCT, serum procalcitonin; CRP, C-reactive protein; Cr, creatinine; BNP, serum brain natriuretic peptide; LVEF, left ventricular ejection fraction; SpO_2_, blood oxygen saturation; DE, diaphragmatic excursion; TFdi, diaphragmatic thickening fraction; TFic, intercostal muscle-thickening fraction; DII, diaphragm-intercostal index.

### Correlation between ultrasound indicators and respiratory mechanics parameters

**Figure 6:**
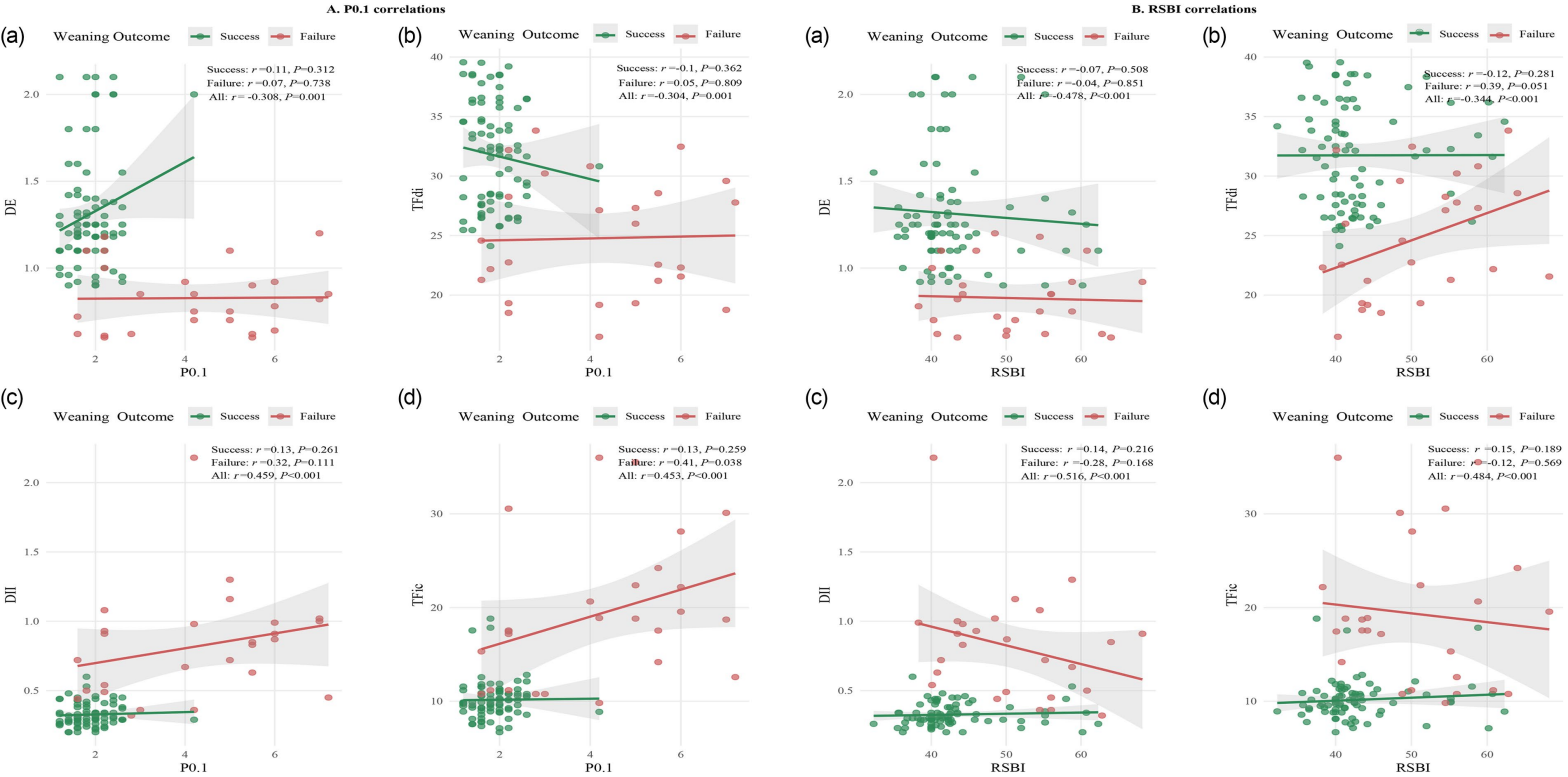
Correlation analyses between ultrasound indicators at 30 min of SBT (pre-extubation) and indicators (P0.1 and RSBI). **A(a)**, correlation between P0.1 and DE at 30 min of SBT in two groups; **A(b)**, correlation between P0.1 and TFdi at 30 min of SBT in two groups; **A(c)**, correlation between P0.1 and DII at 30 min of SBT in two groups; **A(d)**, correlation between P0.1 and TFic at 30 min of SBT in two groups; **B(a)**, correlation between RSBI and DE at 30 min of SBT in two groups; **B(b)**, correlation between RSBI and TFdi at 30 min of SBT in two groups; **B(c)**, correlation between RSBI and DII at 30 min of SBT in two groups; **B(d)**, correlation between RSBI and TFic at 30 min of SBT in two groups; RSBI: rapid shallow breathing index; P0.1, airway occlusion pressure at 100 msec; DE, diaphragmatic excursion; TFdi, diaphragmatic thickening fraction; TFic, intercostal muscle-thickening fraction; DII, diaphragm-intercostal index.

In the total population, P0.1 was significantly correlated with DE, TFdi, TFic, and DII, with correlation coefficients higher than 0.3. P0.1 was inversely linked to DE and TFdi but positively associated with TFic and DII (*p* ≤ 0.001). RSBI was moderately correlated with ultrasound indicators (DE, TFdi, TFic, and DII), with correlation coefficients higher than those of the P0.1 indicator and a direction similar to that of the P0.1 indicator. After grouping, only P0.1 and DII presented a significant positive correlation (r = 0.41, *p* = 0.038) in the extubation failure group (Figure 6).

### Independent risk factors for weaning failure

**Figure 7:**
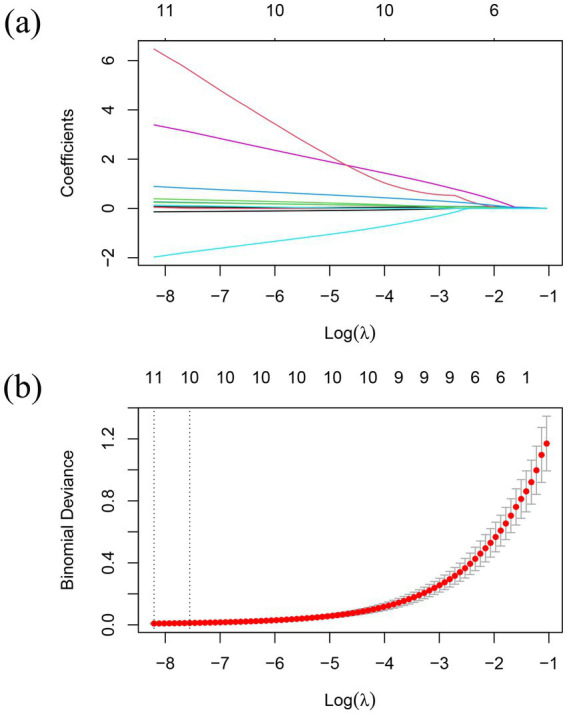
Risk factors screened by the Lasso-logistic regression model. **(a)**, the Lasso coefficient curve of variables, with a vertical line marked at the value obtained through 10-fold cross-validation. With the shrinkage of *λ* value, the degree of model compression is enhanced, and the function of selecting important variables in the model is strengthened; **(b)**, cross-validation results. The value between the two dashed lines refers to the range of positive and negative standard deviations of log (λ).

**Figure 8:**
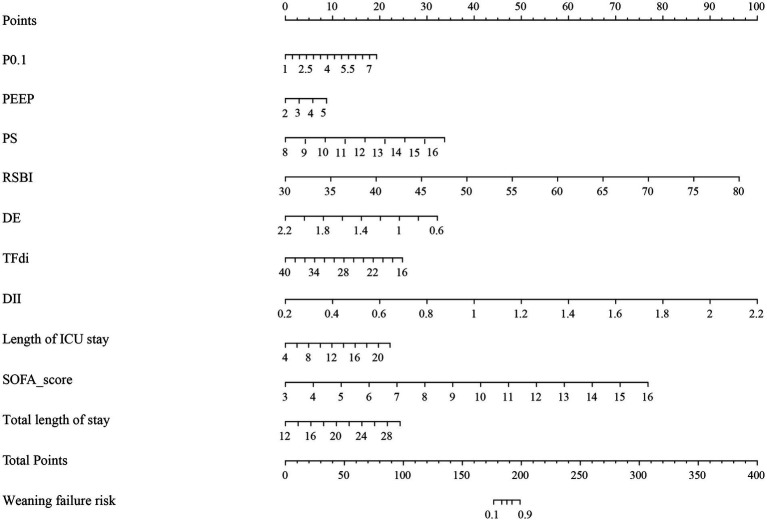
Nomogram for prediction of weaning failure. RSBI: rapid shallow breathing index; P0.1, airway occlusion pressure at 100 msec; PEEP, positive end-expiratory pressure; PS, pressure support; DE, diaphragmatic excursion; TFdi, diaphragmatic thickening fraction; DII, diaphragm-intercostal index.

The changes in the coefficients of variables with significant differences mentioned above and penalty parameter (*λ*, marked by vertical dashed lines) in the Lasso-logistic regression are depicted in Figure 7. At the optimal λ value, 10 risk factors were screened, including P0.1, PEEP, PS, RSBI, DE, TFdi, DII, length of ICU day, SOFA_score, and total length of stay (Table 3). These risk prediction factors are visualized in a nomogram, with each prediction variable corresponding to a scoring axis. Based on patient characteristics, scores were read by the axis of each variable, and a sum of scores, which was projected vertically downwards onto the extubation failure risk axis, was obtained to estimate the probability of extubation failure (Figure 8).

**Table 3:** Indicators screened by the Lasso-logistic regression.

Risk factors	Coefficient
P0.1	0.0826
PEEP	0.0452
PS	0.390
RSBI	0.268
DE	−2.024
TFdi	−0.142
DII	6.369
Length of ICU day	0.244
SOFA_score	0.890
Total length of stay	0.131

RSBI: rapid shallow breathing index; P0.1, airway occlusion pressure at 100 msec; PEEP, positive end-expiratory pressure; PS, pressure support; DE, diaphragmatic excursion; TFdi, diaphragmatic thickening fraction; DII, diaphragm-intercostal index.

### Predictive value of ultrasound indicators for weaning failure

**Figure 9:**
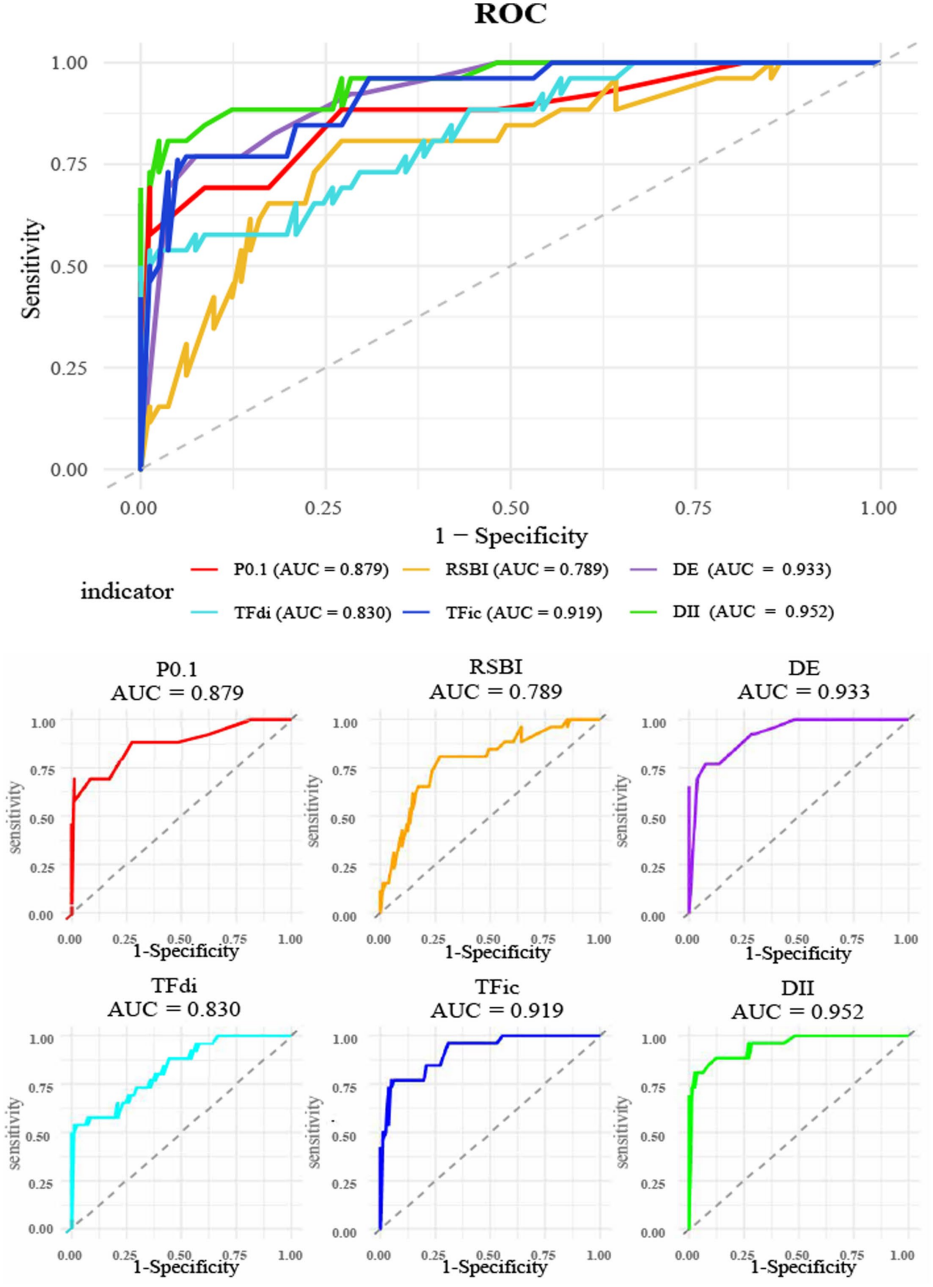
ROC curves of respiratory muscle ultrasound and mechanical indices for predicting weaning failure. RSBI: rapid shallow breathing index; P0.1, airway occlusion pressure at 100 msec; DE, diaphragmatic excursion; TFdi, diaphragmatic thickening fraction; TFic, intercostal muscle-thickening fraction; DII, diaphragm-intercostal index.

We further evaluated the predictive value of different variables for extubation failure using ROC analysis (Table 4; Figure 9). DII showed an AUC of 0.952 (95% CI: 0.907–0.998), significantly higher than RSBI (AUC = 0.789) and TFdi (AUC = 0.830) by DeLong test (*p* = 0.008 and *p* = 0.031, respectively). In the sensitivity analysis excluding the 5 patients with SBT failure, DII’s AUC for true post-extubation failure remained excellent at 0.942 (95% CI: 0.887–0.996), and the DeLong test compared with the primary analysis revealed no significant difference (*p* = 0.779), confirming that the inclusion of SBT failures did not bias the predictive performance.

**Table 4:** Results of ROC curves.

Indicator	AUC	95%CI	Youden_Index	Best_Threshold	Sensitivity	Specificity
DII	0.952	0.907–0.998	0.783	0.485	0.808	0.975
DE	0.933	0.881–0.985	0.695	0.935	0.769	0.926
TFic	0.919	0.860–0.978	0.720	12.575	0.769	0.951
P0.1	0.879	0.792–0.966	0.680	2.700	0.692	0.988
TFdi	0.830	0.738–0.922	0.526	25.020	0.538	0.988
RSBI	0.789	0.687–0.890	0.536	43.400	0.808	0.728

RSBI: rapid shallow breathing index; P0.1, airway occlusion pressure at 100 msec; DE, diaphragmatic excursion; TFic, intercostal muscle-thickening fraction; TFdi, diaphragmatic thickening fraction; DII, diaphragm-intercostal index.

### Repeatability of ultrasound measurements

The intra-observer consistency *ICC* values for Tdi^exp^ and Tic^exp^ measured by the same researcher three times were 0.887 and 0.911, respectively (*p* < 0.001). According to the guideline of reporting *ICC* value for reliability research (21), it was suggested that diaphragm and intercostal muscle ultrasound measurement had good reproducibility (Table 5).

**Table 5:** The repeatability of diaphragm and intercostal muscle ultrasound measurement.

Observer consistency		ICC	95%CI	Reliability level	*p* value
Intra-observer consistency		0.887	0.728–0.950	Good	<0.001
Observer 1stTdi^exp^/mm	1.51 ± 0.31				
Observer 2ndTdi^exp^/mm	1.42 ± 0.40				
Observer 3rdTdi^exp^/mm	1.38 ± 0.27				
Intra-observer consistency		0.911	0.788–0.952	Good	<0.001
Observer 1stTic^exp^/mm	3.68 ± 0.48				
Observer 2ndTic^exp^/mm	3.64 ± 0.40				
Observer 3rdTic^exp^/mm	3.70 ± 0.51				

ICC, interclass correlation coefficient.

## Discussion

This prospective cohort study investigated the predictive value of bedside diaphragm-intercostal muscle ultrasound for extubation outcomes. The major finding was that DII (TFic/TFdi) had excellent performance in predicting weaning failure (AUC 0.952, 95% CI: 0.907–0.998), with a cutoff >0.485 (sensitivity 80.8%, specificity 97.5%), significantly outperforming single diaphragm parameters. Furthermore, in a sensitivity analysis excluding SBT failures, DII remained highly predictive of true post-extubation failure (AUC 0.942), confirming that its predictive capacity is not artificially inflated by including SBT failure as an outcome. These findings provide a more comprehensive perspective for respiratory muscle function assessment in extubation decision-making.

Although transdiaphragmatic pressure is the physiological gold standard for diaphragm function assessment, its invasiveness and complicated operation limit its routine clinical application. In contrast, P0.1, as a non-invasive parameter directly available from a ventilator, can effectively reflect the strength of the respiratory drive (22, 23) as well as extubation outcomes (24–27). Consistently, our study suggested high predictive value of P0.1 [AUC: 0.879, 95% CI: 0.792–0.966]. Similarly, RSBI, a key predictive tool for extubation (28–30), demonstrated good predictive performance in this study [AUC: 0.789, 95% CI: 0.687–0.890], coincident with the finding of Trivedi et al. (31). Of note, this study found that DII and multiple ultrasound parameters (DE, TFdi, TFic) were strongly linked to P0.1 and RSBI, which not only substantiated the reliability of respiratory muscle ultrasound evaluation but also indicated its inherent consistency with traditional respiratory mechanics evaluation in physiological mechanisms.

Diaphragm ultrasound (e.g., DE and TFdi) is widely used to predict extubation outcomes (32–34). However, relying solely on diaphragm evaluation may have limitations (11, 35, 36). Why do traditional indices such as RSBI and diaphragm excursion sometimes fail to accurately predict weaning failure? RSBI reflects the net output of the entire respiratory system but does not distinguish whether tidal volume is generated by a healthy diaphragm or by excessive compensatory recruitment of accessory muscles. Similarly, diaphragm excursion and thickening fraction assess only the primary inspiratory muscle, missing the critical phenomenon of accessory muscle overactivation. DII addresses this gap by directly quantifying the balance between the diaphragm and intercostal muscles (TFic/TFdi), thereby capturing the pathophysiological state of “over-compensation” that precedes overt weaning failure.

The above mechanism is relatively typical in patients with chronic obstructive pulmonary disease (COPD). In COPD patients, lung hyperinflation may lead to geometric changes of the diaphragm and a reduction in contractile efficiency, making the compensatory role of accessory respiratory muscles (particularly the parasternal intercostal muscles) more important. A study by Rittayamai et al. (37) showed that in stable COPD patients, the intercostal muscle thickening fraction (TFic) and its ratio to the diaphragm thickening fraction (TFdi) were both higher than those in healthy controls, and this ratio was negatively correlated with the degree of airflow obstruction (r = −0.67, *p* < 0.001). In that study, COPD patients who experienced moderate-to-severe exacerbations within one year also had a higher TFic/TFdi ratio than those without exacerbations (80.4% vs. 44.0%, *p* = 0.029). These observations suggest that the TFic/TFdi ratio may help quantify the balance between accessory muscle compensation and diaphragm function, and may be associated with clinical prognosis. Based on this background, eligible COPD patients were enrolled in the present study. The accessory-muscle-dependent characteristics of this population provide a reference scenario for testing the clinical applicability of DII. Nevertheless, the predictive value of DII in other populations (e.g., non-COPD ICU patients) remains to be further investigated.

Successful extubation relies on a balance between respiratory load and respiratory drive (38). Impairment of the inspiratory muscles, particularly the diaphragm (39), is a key driver of extubation failure (7, 40–42). Prolonged MV can cause diaphragm atrophy and functional decline (43), which in turn impairs extubation outcomes (44, 45). However, as a whole, the respiratory system mobilizes accessory muscles for compensation when diaphragm function is impaired. Accessory muscle groups, such as the intercostal muscles, are activated early during increased respiratory drive (12, 46), and intercostal muscles are recruited earlier and to a greater extent than the diaphragm (47). Vivier et al. (48) found that evaluating the diaphragm alone was insufficient to guide weaning and that comprehensive evaluation of other accessory muscles is necessary. By integrating diaphragm and intercostal muscles through the DII, this study enhances ultrasound assessment of the physiological status of the respiratory muscles and prediction of extubation failure. We observed that at both 30 min of SBT and 24 h post-extubation, DII and TFic were significantly higher in the failure group than in the success group. This finding supports the notion that in patients with impaired diaphragm function, the parasternal intercostal muscles are significantly recruited to compensate for increased respiratory work, consistent with the findings of Dres et al. (12).

Regarding the use of PEEP 0 cmH_2_O during the SBT: We acknowledge that this setting deviates from current international guidelines, which typically recommend PEEP 5–8 cmH_2_O during SBT. The rationale for our choice was to create a deliberately challenging condition that unmasks latent diaphragmatic dysfunction and compensatory accessory muscle recruitment. This approach maximizes the sensitivity of DII to detect respiratory muscle imbalance. However, using PEEP 0 cmH_2_O increases diaphragmatic afterload and may not reflect routine clinical practice. Therefore, the optimal DII cutoff (>0.485) derived under this specific SBT protocol may not be directly transferable to units using higher PEEP levels. We suggest that future multi-center studies validate DII under guideline-recommended PEEP settings (5–8 cmH_2_O) before broader clinical adoption.

### Limitations

This study has several limitations. First, it is a single-center study with a relatively small sample size, and although we applied strict inclusion/exclusion criteria, the heterogeneity of patient populations and etiologies may limit generalizability. Second, the SBT protocol used PEEP 0 cmH_2_O, which differs from guideline recommendations; as discussed above, this may affect the external validity of the DII cutoff. Third, we excluded obese patients (BMI ≥ 35 kg/m^2^) and those with severe heart failure due to technical limitations (poor ultrasound image quality in obesity) and to minimize confounding (heart failure independently influences weaning outcomes). Consequently, our findings cannot be generalized to these important ICU subpopulations, and future studies specifically targeting obese and heart failure patients are needed. Fourth, we did not assess other accessory respiratory muscles (e.g., sternocleidomastoid, scalene), which may also contribute to compensation during weaning. Fifth, the post-extubation ultrasound data (24 h) were collected for exploratory descriptive purposes only; clinical prediction should be based solely on pre-extubation measurements.

## Conclusion

This study suggests that joint assessment of the diaphragm and intercostal muscles using the DII has potential application value in predicting weaning failure in mechanically ventilated patients. A higher DII value during the extubation preparation period is independently associated with an increased risk of weaning failure. The DII provides a more comprehensive perspective on respiratory muscle function by integrating diaphragm function and intercostal muscle compensatory response. However, extubation outcomes are influenced by multiple factors. Before clinical adoption, the DII requires validation in prospective, multi-center, large-scale studies using standardized SBT protocols (including guideline-recommended PEEP levels) and in populations that were excluded from this study (obesity, heart failure).

## Data Availability

The original contributions presented in the study are included in the article/supplementary material, further inquiries can be directed to the corresponding author.

## Glossary

AUC: Area under the curve
BNP: B-type natriuretic peptide
CI: Confidence interval
Cr: Creatinine
CRP: C-Reactive protein
DE: Diaphragmatic excursion
DII: Diaphragm-Intercostal index
HR: Heart rate
LVEF: Left ventricular ejection fraction
MAP: Mean arterial pressure
OI: Oxygenation index
OR: Odds ratio
P0.1: Pressure at 100 milliseconds after airway occlusion
PCT: Procalcitonin
PEEP: Positive end-expiratory pressure
PS: Pressure support
ROC: Receiver operating characteristic
RSBI: Rapid shallow breathing index
SBT: Spontaneous breathing trial
SpO2: Oxygen saturation
TFdi: Diaphragm thickness fraction
TFic: Intercostal muscle thickness fraction
